# Transcriptional regulation of *Acsl1* by CHREBP and NF-kappa B in macrophages during hyperglycemia and inflammation

**DOI:** 10.1371/journal.pone.0272986

**Published:** 2022-09-02

**Authors:** Prashanth Thevkar-Nagesh, Justine Habault, Maud Voisin, Sophie E. Ruff, Susan Ha, Rachel Ruoff, Xi Chen, Shruti Rawal, Tarik Zahr, Gyongyi Szabo, Inez Rogatsky, Edward A. Fisher, Michael J. Garabedian

**Affiliations:** 1 Department of Microbiology, NYU School of Medicine, New York, NY, United States of America; 2 Department of Medicine, NYU School of Medicine, New York, NY, United States of America; 3 Department of Medicine, Beth Israel Deaconess Medical Center, Harvard Medical School, Boston, MA, United States of America; 4 Department of Urology, NYU School of Medicine, New York, NY, United States of America; 5 Hosptial for Special Surgery, New York, NY, United States of America; 6 Graduate Program in Immunology and Microbial Pathogenesis, Weill Cornell Graduate School for Medical Sciences, New York, NY, United States of America; The Chinese University of Hong Kong, HONG KONG

## Abstract

Acyl-CoA synthetase 1 (ACSL1) is an enzyme that converts fatty acids to acyl-CoA-derivatives for lipid catabolism and lipid synthesis in general and can provide substrates for the production of mediators of inflammation in monocytes and macrophages. *Acsl1* expression is increased by hyperglycemia and inflammatory stimuli in monocytes and macrophages, and promotes the pro-atherosclerotic effects of diabetes in mice. Yet, surprisingly little is known about the mechanisms underlying *Acsl1* transcriptional regulation. Here we demonstrate that the glucose-sensing transcription factor, Carbohydrate Response Element Binding Protein (CHREBP), is a regulator of the expression of *Acsl1* mRNA by high glucose in mouse bone marrow-derived macrophages (BMDMs). In addition, we show that inflammatory stimulation of BMDMs with lipopolysaccharide (LPS) increases *Acsl1* mRNA via the transcription factor, NF-kappa B. LPS treatment also increases ACSL1 protein abundance and localization to membranes where it can exert its activity. Using an *Acsl1* reporter gene containing the promoter and an upstream regulatory region, which has multiple conserved CHREBP and NF-kappa B (p65/RELA) binding sites, we found increased *Acsl1* promoter activity upon CHREBP and p65/RELA expression. We also show that CHREBP and p65/RELA occupy the *Acsl1* promoter in BMDMs. In primary human monocytes cultured in high glucose versus normal glucose, ACSL1 mRNA expression was elevated by high glucose and further enhanced by LPS treatment. Our findings demonstrate that CHREBP and NF-kappa B control *Acsl1* expression under hyperglycemic and inflammatory conditions.

## Introduction

Atherosclerosis is a chronic inflammatory disease characterized by infiltration of monocytes, their differentiation into macrophages, and the accumulation of lipid-laden macrophages, termed “foam cells," in the arterial wall forming plaques [1]. In atherosclerosis progression, circulating monocytes continue to be recruited to the plaque by inflammatory signals and become activated macrophages, further contributing to the vascular pathology.

Several factors drive the inflammatory response of macrophages, including ACSL1, which converts long-chain fatty acids into acyl-CoA derivatives [2, 3]. While the preferred substrates for ACSL1 are multiple fatty acids [4], in monocytes/macrophages, ACSL1 also appears to use arachidonic acid as a specific substrate to produce prostaglandin E2 (PGE2) to promote inflammation [3, 5, 6].

ACSL1 has emerged as a mediator of enhanced atherosclerosis associated with diabetes [3] by accelerating the progression of atherosclerosis [7]. There is an increase in the expression of *Acsl1* mRNA in monocytes from human diabetic patients and from mouse models of type-1 diabetes [5]. Moreover, there was increased expression of Acsl1 when macrophages were cultured under diabetes-relevant high glucose (25 mM) compared to normal glucose (5.5 mM), suggesting that the effect of hyperglycemia on *Acsl1* expression is cell-autonomous [5]. Significantly, mice lacking *Acsl1* in monocytes and macrophages prevented the accelerated progression of atherosclerosis in diabetes [5]. Thus, ACSL1 is a crucial regulator of the pro-atherosclerotic effects of diabetes. Consistent with ACSL1 links to cardiovascular and metabolic diseases in humans, analysis of genome-wide association studies found intronic SNPs in ACSL1 associated with atherosclerosis and type-2 diabetes [8].

In addition to hyperglycemia, *Acsl1* expression in macrophages is induced by inflammatory stimuli, such as lipopolysaccharide (LPS) and gram-negative bacteria (E. coli) [9]. ACSL1 protein expression and localization to membranes are also increased in inflammatory (M1) macrophages relative to non-activated (M0) macrophages, presumably to place ACSL1 enzymatic activity in proximity with its substrates as part of its proinflammatory response [2].

Despite the increased expression of *Acsl1* in macrophages by hyperglycemic and inflammatory stimuli, the mechanisms mediating the transcriptional regulation of *Acsl1*, including the transcription factors controlling *Acsl1* expression, are incompletely characterized. Here we report that transcriptional regulation of *Acsl1* stimulated by high glucose and inflammatory stimuli involves CHREBP and NF-kappa B.

## Materials and methods

### Cell culture

Human embryonic kidney (HEK) 293 cells (ATCC) were cultured in Dulbecco’s modified Eagle’s medium (DMEM, Corning) containing 10% fetal bovine serum (FBS, Hyclone) and 1% PenStrep (100 U/mL Penicillium and 100ug/mL Streptomycin) in either 4.5 g/L D-glucose (HG) or 1 g/L D-glucose + 3.5 g/L L-glucose (Sigma) (NG) to serve as an osmotic control. Cells were tested for mycoplasma and were negative. Cells were cultured in 5% CO_2_ at 37°C.

### Animals

We obtained wild-type mice (C57B16J) from Jackson labs. Dr. Claudia Han from the Christopher Glass lab at UCSD kindly provided tibias and femurs from *Mlxipl* deficient mice *(Chrebp*^***-/-***^*)*. The animals were cared for per the National Institutes of Health guidelines and the NYU School of Medicine and the UCSD Institutional Animal Care and Use Committees. Mice were euthanized by CO_2_ followed by cervical dislocation following approved guidelines for the euthanasia of animals.

### Preparation of bone marrow-derived macrophages (BMDMs)

BMDMs were isolated from the tibias and femurs of 6-12-week-old male wild-type C57BL6J and *Chrebp*^***-/-***^ mice. Isolated bone marrow cells were treated with red blood cell lysis buffer (Sigma) and re-suspended in differentiation medium (DMEM and L-glutamine with 1 g/L D-glucose + 3.5 g/L L-glucose (normal glucose; NG), or 4.5 g/L D-glucose (high glucose; HG), supplemented with 20% FBS and 10 ng/μL macrophage colony-stimulating factor (M-CSF) (PeproTech, Inc., Rocky Hill, NJ). Cells were passed through a 70μm filter to clear debris. Cells were then plated in 10cm non-tissue coated plates and allowed to differentiate in either NG or HG-containing media for 7 days to obtain non-activated (M0) macrophages. On day 7, the cells were washed in PBS, re-plated at the desired cell density in a 6-well dish in normal and high glucose media, and allowed to attach to the plate. We treated cells with LPS (10ng/ml) for the indicated times and isolated either RNA or protein. For some experiments, cells were pretreated for 4 hours with the NF-kappa B inhibitor, caffeic acid phenethyl ester (CAPE [5μM]) before LPS treatment.

### Promoter motif prediction

We used the Eukaryotic Promoter Database for predicting the putative CHREBP (*Mlxipl)* and NF-Kappa B (p65/RELA) binding sites, as well as PPAR gamma and SREBP2 (*Srebf2*) sites on the mouse and human ACSL1 upstream regulatory regions and promoters [10].

### Luciferase assay

Following the manufacturer’s protocol, we transfected HEK293 cells (24-well format) with Lipofectamine 3000 (Invitrogen). For co-transfection experiments, plasmids expressing NF-Kappa B (p65/RELA) (150ng) and CHREBP (100, 150, 200, 250ng) were co-expressed with 250ng of *Acsl1*-Gaussia luciferase (*Acsl1*-GLuc) reporter plasmid. This reporter construct contains regions 1466 bp upstream and 216 bp downstream from the start site of transcription for the mouse *Acsl1* gene fused to the Gaussia luciferase gene (GeneCopoeia; # MPRM39476). Gaussia Luciferase possesses a natural secretory signal and is secreted into the cell culture media upon expression. Therefore, cell lysis is not necessary for assaying the expression levels of this bioluminescent enzyme. We adjusted the total DNA to 750ng with an empty expression vector. At 48 hours post-transfection, we collected the media containing the Gaussia Luciferase enzyme, and luciferase activity was measured following the manufacturer’s protocol (GL-S buffer GeneCopoeia; #LF061). We used a separate set of transfected cells for protein expression studies by western blot. Luciferase activity was measured using the LMax microplate reader luminometer with an integration time of 3 sec. The pcDNA3 Flag-p65/RelA expression vector was purchased from Addgene (plasmid #20012) and deposited by Stephen Smale (UCLA) [11]. The N-terminal Myc-tagged CHREBP expression vector was purchased from Addgene (plasmid #39235) and deposited by Isabelle Leclerc (University of Bristol) [12].

### RNA isolation, cDNA synthesis, and qPCR

We isolated total RNA using RNeasy Mini Kit (Qiagen) and included an On-column DNase digestion step during the isolation process. cDNA was synthesized from 500ng of RNA using Thermo Scientific™ Verso cDNA Synthesis Kit (AB1453B) following the manufacturer’s instructions. Quantitative real-time PCR was performed on the QuantStudio 6 Flex (Applied Biosystems) instrument using SYBR Green Fast Master Mix (Applied Biosystems). We used 5 ng of cDNA and 100nM of primers for the qPCR reaction. Gene expression was calculated using the relative quantification (2^−ΔΔC^T method) or by quantification of absolute *Acsl1* transcript copy number using a standard curve generated with known quantities of mouse *Acsl1* cDNA.

### Primers

mouse *Acsl1* mRNA

F 5’-GCGGAGGAGAATTCTGCATAGAGAA-3’;

R 5’- ATATCAGCACATCATCTGTGGAAG-3’

mouse Cyclophilin A1 mRNA

F: 5’-GGCCGATGACGAGCCC-3’

R: 5’-TGTCTTTGGAACTTTGTCTGCAA-3’

mouse *Acsl1* hnRNA

F: 5’-TCACTCCTTATCACCTCTTC-3’

R: 5’-CTCCAGAGCTTTGAGGCTGATG -3’

mouse *Acsl1* upstream regulatory region (numbering from the transcription start site); ChIP

5’-GGACACTGAGCAACAGTGATGGC-3’

-1075 R: 5’-GCCCATGCCTGTCACAAAGC-3’

Human ACSL1 mRNA

F 5’-CTTATGGGCTTCGGAGCTTTT-3’

R 5’-CAAGTAGTGCGGATCTTCGTG-3’

Human 18S RNA

F: 5’-GTAACCCGTTGAACCCCATT-3’

R: 5’-CCATCCAATCGGTAGTAGCG-3’

### Cell fractionation

For cellular fractionation studies from BMDMs, we used the Subcellular Protein Fractionation Kit for Cultured Cells (Thermo Fisher; #78840). This kit uses hypotonic lysis of cells, differential centrifugation, and detergent extraction to fractionate the BMDMs into cytoplasmic and membrane fractions for western blot. This kit captures proteins in both internal membranes excluding nuclear membrane and plasma membranes. We purified mitochondria from BMDMs using the Mitochondria Isolation Kit for Cultured Cells (Thermo Fisher; # 89874). This kit uses a proprietary formulation to lyse cells and differential centrifugation to separate the mitochondrial fraction for western blot.

### Immunoblotting

Cells were lysed in lysis buffer (50 mM Tris-HCl [pH 7.4], 150 mM NaCl, 0.5% sodium dodecyl sulfate [SDS], 0.5% sodium deoxycholate, 1% Triton X-100, and 1× protease inhibitor cocktail [Roche]), and the total amount of protein was quantified using Pierce™ Rapid Gold BCA Protein Assay Kit (Thermo Scientific). Equal amounts of proteins were resolved on 10 or 15% Tris-glycine SDS-PAGE under reducing conditions and transferred onto Immobilon-P Membrane, PVDF, 0.45 μm (Millipore). Membranes were probed with rabbit anti-ACSL1 (#9189, 1:1,000; Cell Signaling or # PA5-17136, 1;1000; Thermo Scientific), rabbit anti-CHREBP Antibody (# NB400-135, 1:500; Novus Biologicals), rabbit anti-histone H3 (1:1,000; Cell Signaling), rabbit anti-actin (1:5,000; Abcam; ab8227), rabbit-anti Sodium Potassium ATPase antibody (1:5,000; Abcam, ab76020) or mouse anti-tubulin Mouse Monoclonal Antibody (HRP-66031, 1:5000, Proteintech), followed by horseradish peroxidase-conjugated anti-mouse, or anti-rabbit IgG antibody (1:5,000; Life Technologies). We visualized the protein bands using Clarity Western ECL Substrate (BioRad) and acquired the images on an Odyssey Fc imaging system (LI-COR).

### Immunofluorescence of CHREBP from BMDMs

BMDMs were fixed in 4% methanol-free paraformaldehyde (Fisher Scientific) and permeabilized with 0.2% Triton X-100. We used 5% mouse or rabbit serum for blocking. Cells were stained with rabbit anti-CHREBP antibody (# NB400-135, 1:100; Novus Biologicals), followed by Alexa 488-conjugated goat anti-rabbit IgG secondary antibody (1:400; Invitrogen) for 1 hour at room temperature. Cells were stained with the DNA-binding dye Hoechst (5 μg/mL; Invitrogen), and coverslips were mounted in a mounting medium (Sigma-Aldrich). Fluorescent images were acquired by sequential scanning on a Leica SP5 confocal laser scanning microscope.

### ChIP-qPCR

BMDMs were cultured in high glucose media with or with LPS (10ng/ml, 1 hour treatment). Cells were double cross-linked with 2mM DSG (ProteoChem; # c1104) in PBS for 20 min and 1% formaldehyde for 10 min. Crosslinking was quenched with Tris-HCl pH 7.5. Cells were collected, washed with PBS, and cell pellets snap-frozen with liquid nitrogen. Cell pellets were resuspended in nuclei isolation buffer (50 mM Tris-HCl pH 8.0, 60 mM KCl, 0.5% NP40), nuclei collected, and resuspended in sonication buffer (RIPA buffer). Samples were sonicated in TPX PMP tubes (Diagenode) using Diagenode Bioruptor Pico sonicator for 10min (30s on, 30s off, x10 cycles). Inputs (2%) were collected, and supernatants were then incubated overnight with antibodies to CHREBP (Novus; Rabbit, # NB400-135), p65 (Cell Signaling; Rabbit, # 8242), or normal rabbit IgG (Sigma Aldrich, # 12–370) pre-incubated with Protein A and Protein G Dynabeads (Invitrogen). Immunocomplexes were then washed and cross-linking reversed overnight at 65°C with 5M NaCl. DNA was isolated with the Zymo ChIP DNA Clean and Concentrator kit (Zymo Research, # D5205). qPCR was performed as described above, with primers targeting the mouse *Acsl1* upstream regulatory region between -1251 to -1075. Relative enrichment was calculated as a percentage of input.

### Human monocyte isolation

Healthy human subjects without preexisting medical conditions provided informed consent for peripheral blood collection at Beth Israel Deaconess Medical Center under IRB protocol # 2019C000971. The blood was mixed in 1:1 with PBS, and monocytes were isolated as described [13]. Monocytes were plated on a 6-well dish (2x10^6^ cells/well), and cultured in RPMI with 10% FBS and 1% penicillin/streptomycin for 24 hours in either 25mM D-glucose or 5mM D-glucose (+20mM L-glucose to control for osmolarity) to mimic diabetic or nondiabetic conditions, respectively. The monocytes were untreated or treated with LPS (10ng/ml) for 24 hours. Total RNA was extracted using a QIAGEN kit and cDNA was synthesized. qPCR was performed to determine the expression of *ACSL1* mRNA relative to 18S RNA.

### Statistical analysis

All statistical analyses were performed using Prism 9 (GraphPad). P-values were calculated using an unpaired t-test for pairwise data comparisons or one-way analysis of variance (ANOVA) for data comparisons of two or more independent groups. A p-value of ≤0.05 was considered significant.

## Results

### Transcriptional induction of *Acsl1* mRNA in macrophages by hyperglycemia

**Fig 1:**
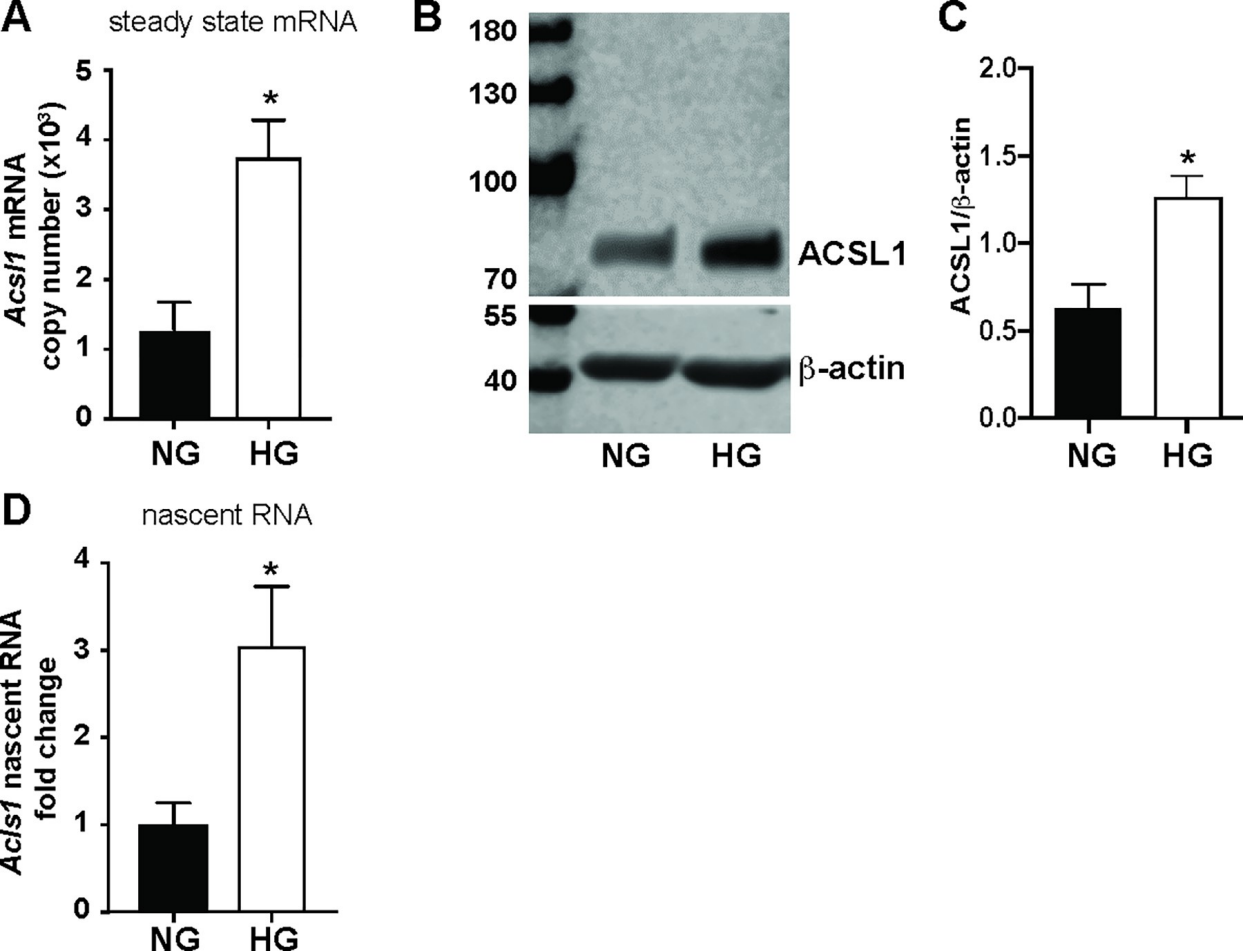
*Acsl1* expression is upregulated in macrophages by HG. A) BMDMs were differentiated under normal glucose (NG) and high glucose (HG), and *Acsl1* mRNA absolute copy number was determined by quantitative real-time PCR (qPCR) using a standard curve generated with known quantities of mouse *Acsl1* cDNA. B) Western blot of total cell lysates from BMDMs cultured in NG and HG using antibodies against ACSL1 and β-actin as (loading control). C) The ratio of ACSL1/β-actin band intensities was quantified using Image studio 5.2 (n = 3). D) Nascent *Acsl1* RNA expression in BMDMs under NG and HG conditions was determined by qPCR. Nascent *Acsl1* expression was quantified relative to cyclophilin A and shown as fold change. The data presented are the means ± standard error (n = 3); the p-value was calculated using the student’s t-test. (*p < 0.05).

It has been reported that the expression of *Acsl1* is upregulated in monocytes and macrophages under diabetic conditions *in vitro* and *in vivo in mice*, and in clinical samples [5, 8, 14]. However, the mechanism whereby *Acsl1* is upregulated by hyperglycemia is not understood. To determine whether *Acsl1* expression is upregulated by diabetes-relevant high glucose, we differentiated primary mouse bone marrow-derived macrophages (BMDMs) under normal glucose (NG; 5.5 mM glucose) or high glucose (HG; 25 mM glucose) conditions. To control for the effects of osmolarity, we supplemented the low glucose media with non-metabolizable L-glucose. We found that *Acsl1* mRNA (Fig 1A) and protein (Fig 1B and 1C) were upregulated under HG compared to NG. To ensure that the increase in *Acsl1* expression is a direct effect of HG rather than an indirect effect of differentiation in HG, we differentiated BMDMs in NG, then switched the cells to HG for 24 hours and measured *Acsl1* expression. We also found an increase in *Acsl1* expression upon acute HG treatment similar to that observed in cells differentiated in chronic HG (S1 Fig). Thus, HG upregulates *Acsl1* expression in BMDMs independent of the glucose concentration during differentiation.

To determine whether the increase in *Acsl1* expression in HG reflected increased transcription, we measured heteronuclear or nascent RNA levels as a surrogate for newly synthesized *Acsl1* transcripts [15]. We found that the increase in steady-state *Acsl1* mRNA was associated with a corresponding increase in the nascent RNA levels under HG as compared to NG conditions (Fig 1D). Thus, hyperglycemia induces the transcription of the *Acsl1* gene in BMDMs.

### CHREBP regulates *Acsl1* transcription under hyperglycemic conditions

CHREBP is a glucose-responsive transcription factor that regulates metabolic genes, including those involved in lipolysis and glycolysis [16–18]. Whereas low glucose concentrations restrain the transcriptional activity of CHREBP via inhibition of the transactivation domain through interaction with a low-glucose inhibitory domain and cytoplasmic sequestration, an increase in intracellular glucose levels relieves this inhibition and promotes CHREBP nuclear localization and binding to DNA sequences termed CHREBP response elements (ChoRE) to drive the expression of glucose-responsive genes [19]. Elevated glucose levels in diabetes increase CHREBP transcriptional activity in liver and adipose tissues [20]. In addition, a ChIP-seq study for CHREBP from white adipose tissue in fasted versus fed mice showed that CHREBP occupied multiple sites upstream of the *Acsl1* transcription start site [21], suggesting that *Acsl1* is a potential target of CHREBP. Therefore, we hypothesized that CHREBP upregulates *Acsl1* expression in macrophages in response to hyperglycemia.

We first determined the cellular localization of CHREBP in BMDMs cultured under NG and HG conditions. We observed increased CHREBP nuclear localization under HG compared to NG conditions by cell fractionation and immunofluorescence (S2 Fig), confirming the suggestion that CHREBP is a potential transcriptional activator of *Acsl1* under HG conditions.

**Fig 2:**
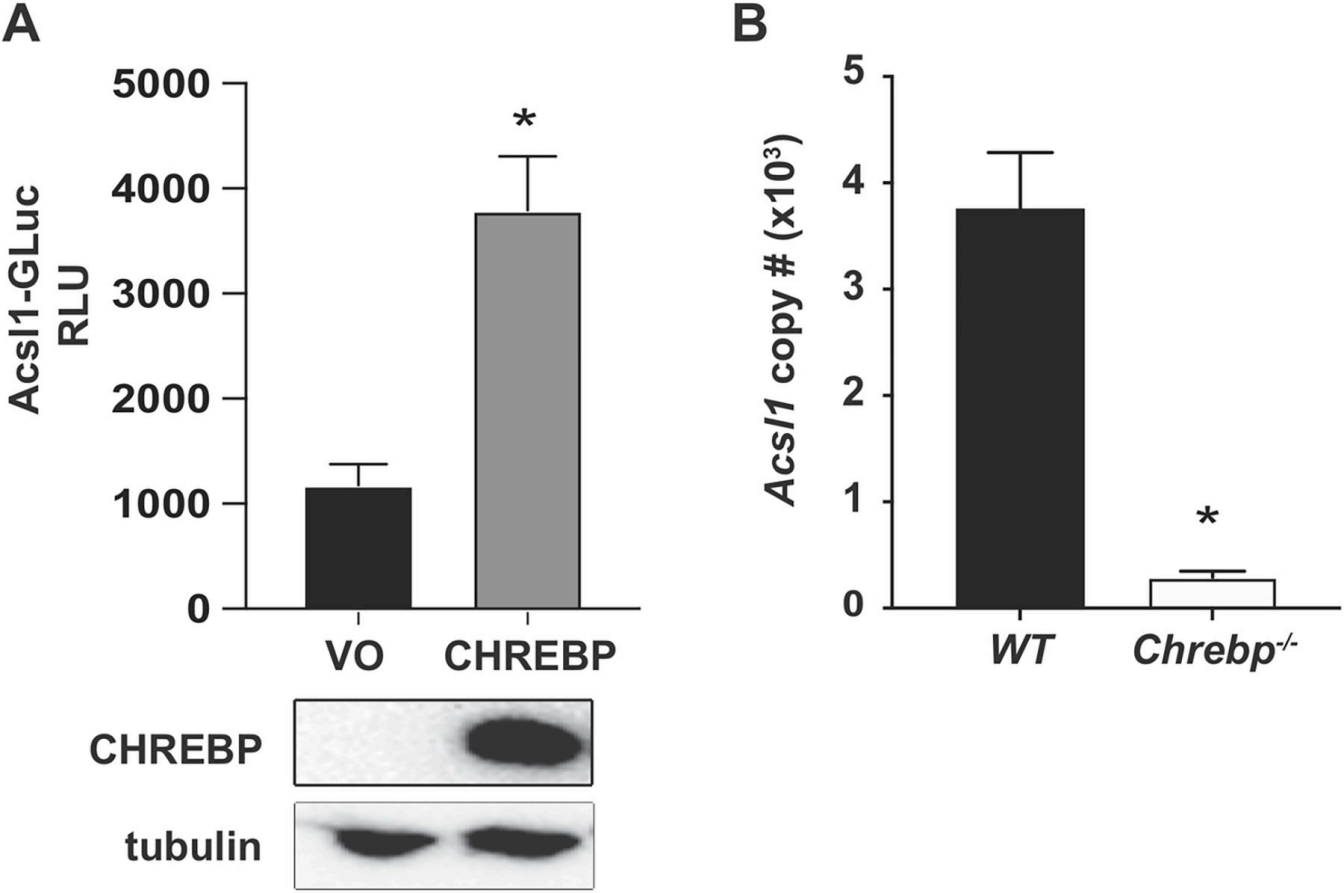
CHREBP contributes to transcriptional upregulation of *Acsl1* in HG. A) CHREBP expression plasmid or vector only (VO) were co-transfected with *Acsl1*-GLuc reporter in HEK293 cells cultured under HG conditions. Luciferase assay was performed 48 hours post-transfection and shown as relative luciferase units (RLU). Expression of transfected Myc-tagged CHREBP is shown by western blot using a c-Myc antibody, with tubulin as a loading control. B) Wild type or *Chrebp*^***-/-***^ BMDMs were differentiated under HG conditions. *Acsl1* mRNA absolute copy number was quantified by qPCR. The data presented are means ± standard error (n = 3); the p-value was calculated using student’s t-test. (*p < 0.05).

We next determined whether overexpression of CHREBP could regulate *Acsl1* promoter activity in a cell-based reporter assay. We measured *Acsl1* promoter activity by performing luciferase assays in HEK293 cells transfected with an *Acsl1* reporter construct, containing the *Acsl1* promoter and ~1.5 kb of an upstream regulatory region fused to the Gaussia luciferase gene (*Acsl1*-GLuc), along with a CHREBP expression construct, or an empty expression vector. We found that *Acsl1* promoter activity was increased in cells overexpressing CHREBP (Fig 2A). Therefore, ectopic expression CHREBP can induce the expression of *Acsl1* in a cell-based reporter assay.

We also evaluated whether endogenous *Acsl1* mRNA expression is reduced by deletion of CHREBP using macrophages from *Chrebp*^***-/-***^ mice [18]. We differentiated BMDMs from wild-type littermate and *Chrebp*^***-/-***^ mice in HG and measured *Acsl1* mRNA expression. We found a marked reduction in *Acsl1* mRNA copy number in *Chrebp*^***-/-***^ compared to wild-type BMDMs (Fig 2B). Thus, CHREBP contributes to endogenous *Acsl1* expression in HG in BMDMs.

### Lipopolysaccharide (LPS) stimulates *Acsl1* expression via NF-kappa B

*Acsl1* expression is also induced by inflammatory stimuli, such as LPS [9], and is an important mediator of the inflammatory response in monocytes and macrophages [22], presumably by providing arachidonic acid to be converted to PGE2 [5]. It has also been reported that individuals with diabetes have higher levels of *ACSL1* mRNA in circulating inflammatory monocytes [5]. Moreover, in a mouse model, myeloid cell-specific deletion of *Acsl1* decreased the expression of proinflammatory cytokines under diabetic conditions [5]. Although it is evident that multiple transcription factors, including PPAR gamma [23] and SREBP2 [24], are capable of upregulating *Acsl1* mRNA, transcription factors facilitating the induction of *Acsl1* in response to inflammatory and hyperglycemic signals have not been completely characterized.

To address the regulation of *Acsl1* by inflammatory signals, we determined the kinetics of *Acsl1* induction by LPS in BMDMs. Since LPS signals via Toll-like receptors (TLRs) to activate NF-kappa B, we compared *Acsl1* to established LPS/NF-Kappa B-responsive genes, *Tnf* and *Il6*. [25, 26]. Whereas LPS induced *Tnf* and *Il6* by 0.5 and 1 hour, respectively, the induction of *Acsl1* in BMDMs became apparent 3 hours post LPS stimulation, peaking at 24 hours (S3 Fig). These findings suggest a different temporal requirement for LPS to induce expression of *Acsl1* compared to *Tnf* and *Il6* [27].

**Fig 3:**
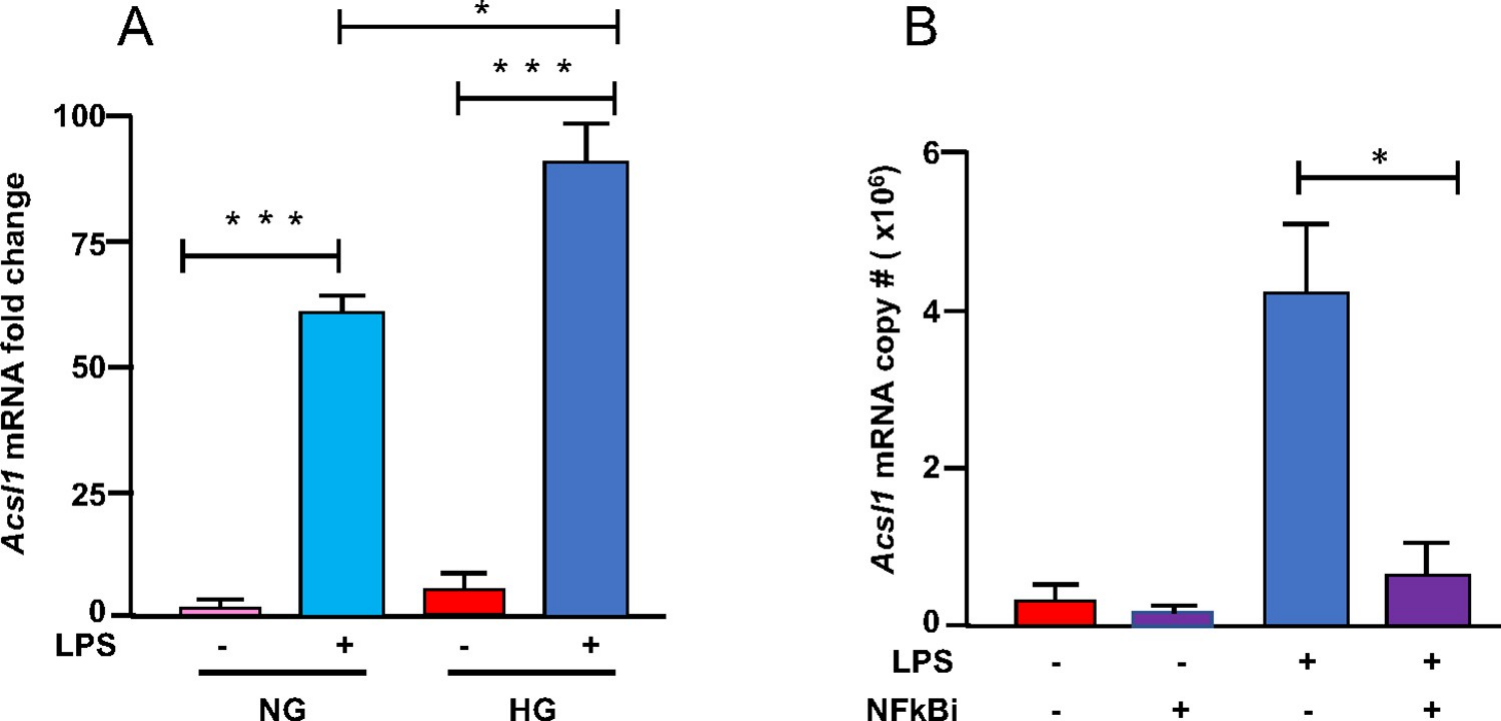
LPS induction of *Acsl1* expression via NF-kappa B. A) BMDMs were differentiated under NG and HG conditions. Cells were stimulated with LPS (10ng/mL) for 24 hours. Total RNA was isolated, and *Acsl1* mRNA was determined by qPCR relative to cyclophilin A1 and shown as fold change. NG treated sample (M0) was set to 1. B) BMDMs were differentiated in HG. Cells were pretreated for 4 hours with NF-kappa B inhibitor CAPE (5 μM) and then treated with LPS for 16 hours. RNA was isolated, and *Acsl1* mRNA copy number was determined by qPCR. The data presented are means ± standard error (n = 3); the p-value was calculated using one-way ANOVA (p < 0.05; **p < 0.01; and ***p < 0.001).

We also determined *Acsl1* expression in macrophages upon combining LPS and HG. In NG, LPS stimulation of macrophages for 24 hours (which results in M1-activated inflammatory macrophages) induced *Acsl1* expression ~60 fold as compared to BMDMs not stimulated by LPS (M0 macrophages) (Fig 3A). Intriguingly, in HG, LPS exposure increased *Acsl1* expression even further (~85-fold). This additional enhancement of *Acsl1* expression by HG and LPS was not observed for *Tnf* or *Il6* (not shown), suggesting *Acsl1* expression is responsive to both inflammatory and glucose signals. Consistent with this are the findings from human monocytes cultured in NG and HG with and without LPS treatment. The expression of *ACSL1* mRNA was increased by HG and LPS, with the highest expression in cells treated with both LPS and HG, lending relevance to our findings to humans (S4 Fig).

We next determined whether NF-kappa B plays a role in the LPS-dependent induction of *Acsl1* by inhibiting NF-kappa B using caffeic acid phenethyl ester (CAPE), which blocks NF-kappa B binding to DNA [28]. BMDMs cultured in HG were treated with either LPS (M1) or left untreated (M0) in the presence or absence of CAPE. CAPE treatment reduced LPS-dependent induction of *Acsl1* expression in macrophages, whereas inhibiting NF-kappa B in the absence of LPS did not affect the expression of *Acsl1* (Fig 3B). Therefore the acquisition of an inflammatory phenotype contributes to *Acsl1* expression.

**Fig 4:**
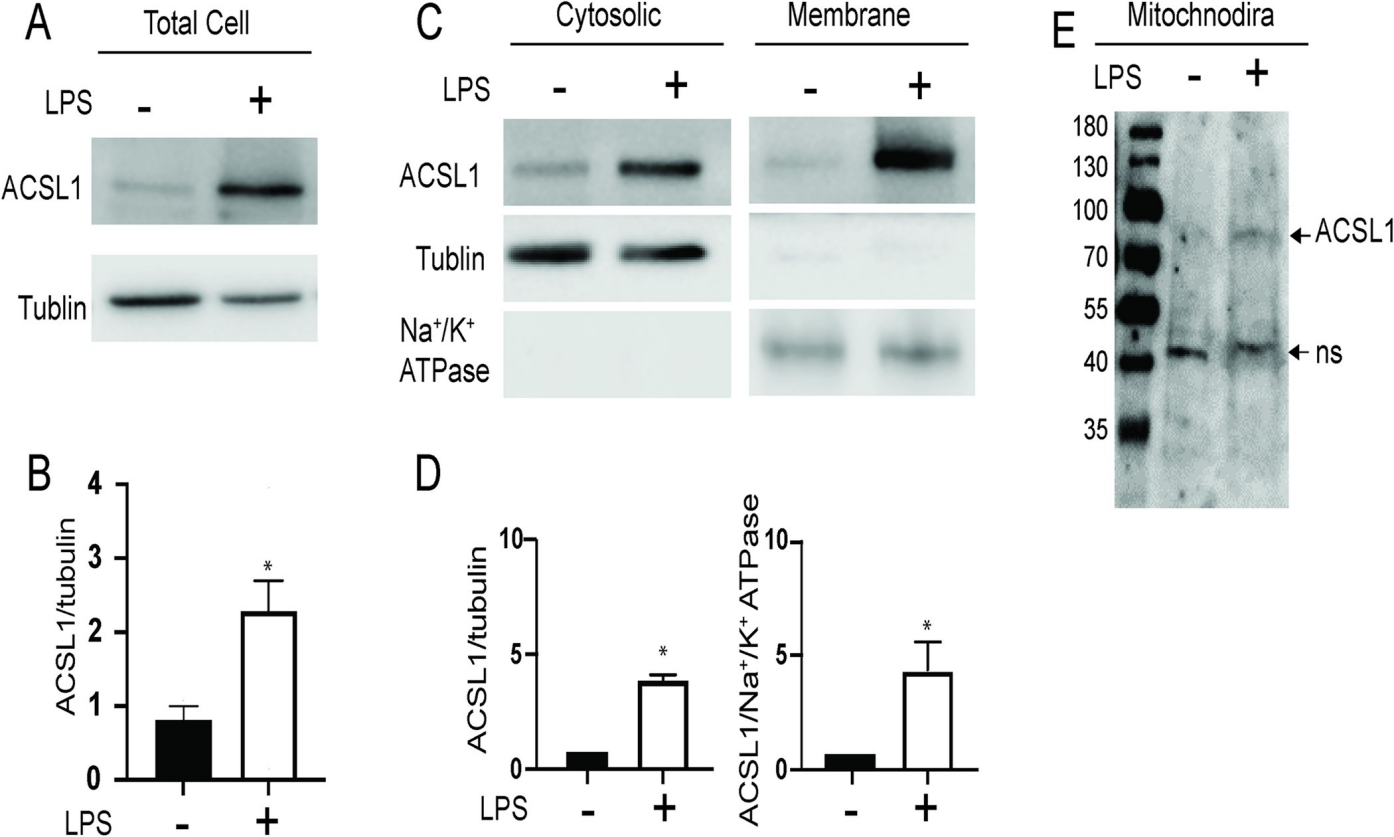
ACSL1 protein abundance and membrane localization increase under inflammatory conditions. BMDMs were differentiated under NG conditions. A) Total cell lysates were prepared with (+) and without (-) LPS treatment (10 ng/ml for 24 hours). ACSL1 protein expression was determined by western blot with an anti-ACSL1 antibody. An anti-alpha tubulin antibody was used as a loading control. B) Bands were quantified using the Image studio 5.2. C) Cytoplasmic and membrane proteins were isolated to determine the localization of ACSL1 protein by western blotting as a function of LPS treatment. Alpha-tubulin (cytoplasmic protein) and Na+/K+ ATPase (membrane-associated protein) were used to confirm the fidelity of fractionation. D) Images were quantitated as in B with cytoplasmic ACSL1 protein normalized to tubulin and membrane ACSL1 protein normalized to Na+/K+ ATPase. The data presented are means ± standard error (n = 3); the p-value was calculated using student’s t-test. (*p < 0.05). E) Mitochondria were isolated from BMDMs, and ACSL1 abundance was determined by western blot with and without LPS treatment. The non-specific (ns) band serves as a loading control.

Increased *Acsl1* mRNA expression upon LPS treatment was also associated with higher ACSL1 protein abundance (Fig 4A and 4B) and localization to membranes (Fig 4C–4E), consistent with published reports [5]. This suggests that inflammatory signals contribute to ACSL1protein expression and membrane localization.

### CHREBP and p65/RELA enhance *Acsl1* promoter activity

Given that both NF-kappa B and CHREBP appear to modulate *Acsl1* expression, we examined the 2 kb upstream regulatory region of the mouse and human *Acsl1* genes for putative ChoRE (binding site for CHREBP) and p65/RELA (binding site for NF-kappa B) sites using the Eukaryotic Promoter Database [10] to predict transcription factor binding sites [29]. We identified multiple CHREBP and p65/RELA binding sites near one another and conservation of these sites between the mouse and human genes (S5 Fig). Such conservation is suggestive of the importance of the sites in transcriptional regulation [30]. In addition, binding motifs of previously reported regulators of ACSL1, including PPAR gamma and SREBP2 [24], were conserved in the promoters of the mouse and human ACSL1 genes (Table 1).

**Table 1:** Prediction of transcription factor motifs 2 kb upstream of the mouse and human ACSL1 gene.

	CHREBP	p65/RELA	PPAR gamma	SREBP2
*Acsl1* mouse	-1784,-1525, -1345,-1136, -945, -905, -639	-1130,-881	-414,-35	-1525,-1345, -617,-341,-306
*ACSL1* human	-1616, -1487, -1171–645, -175,	-1923,-1908, -957,-232	-793,-481	-592,-375,-237, -9,+74

The p-value used for the motif prediction was p<0.001.

**Fig 5:**
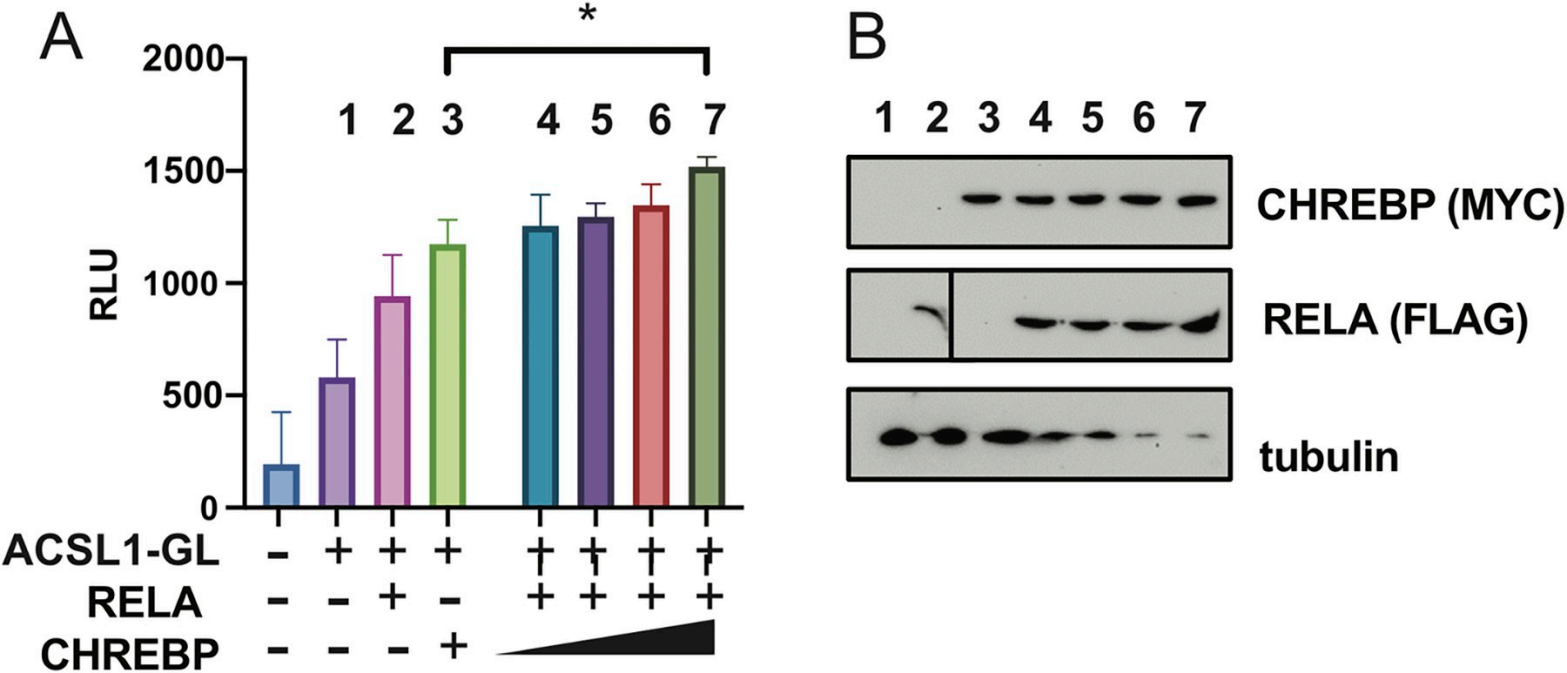
CHREBP and NF-kappa B increase *Acsl1* transcriptional activity. HEK293 cells cultured in HG conditions were transfected with *Acsl1*-GLuc reporter (250ng; columns 1–7) and individually with p65/RELA (150ng; column 2) or CHREBP (150ng; column 3). Cells were transfected with *Acsl1*-GLuc reporter and a fixed amount of p65/RELA (150ng; columns 4–7), along with increasing amounts of CHREBP (100ng, 150ng, 200ng, 250ng; columns 4–7, respectively). Total DNA was adjusted to 750ng with vector only. Luciferase assay was performed 48 hours post-transfection and shown as relative luciferase units (RLU). The data presented are means ± standard errors (n = 3); the p-value was calculated using one-way ANOVA. *p < 0.05. B) Western blot of lysates from panel A with antibodies against the Myc-tag on CHREBP, FLAG-tag on p65/RELA, and tubulin as a loading control. The p65/RELA blot for lanes 1–2 was run on a different gel than lanes 3–7 and denoted by a black line between lanes 2 and 3. Exposure times were the same.

To test the functional significance of NF-kappa B and CHREBP in enhancing *Acsl1* gene expression, we transfected HEK293 cells with *Acsl1*-GLuc, with a fixed amount of the NF-kappa B subunit p65/RELA and increasing amounts of CHREBP, and measured A*csl1* promoter activity. This approach has been used to reveal cooperativity between transcription factors [31]. Co-expression of CHREBP and p65/RELA showed increased A*csl1* promoter activity relative to either transcription factor alone (Fig 5). These results support that CHREBP and NF-kappa B cooperate to increase *Acsl1* transcriptional activity.

### CHREBP and p65/RELA occupy the *Acsl1* promoter

**Fig 6:**
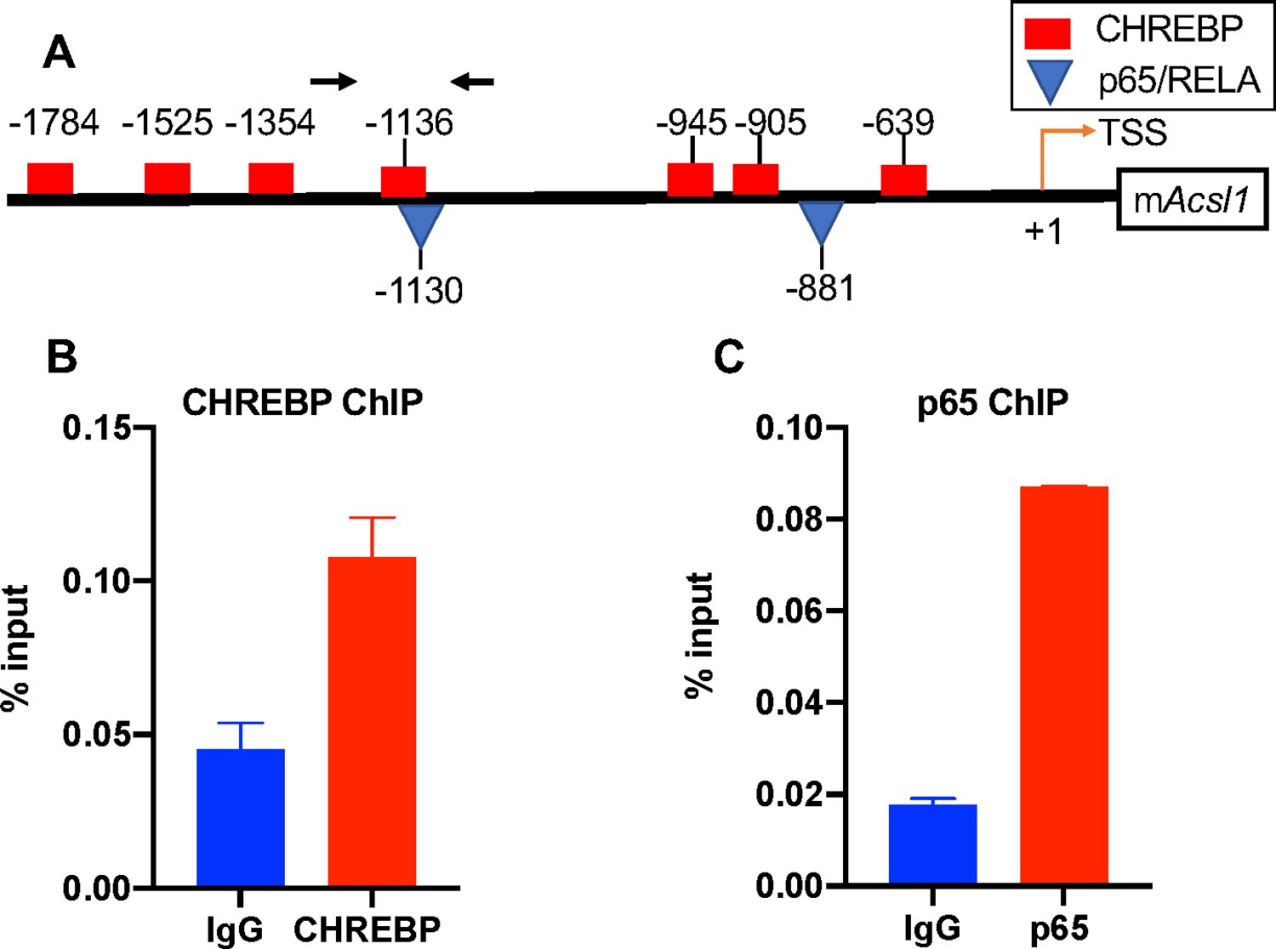
CHREBP and p65/RELA occupy the mouse *Acsl1* gene. A) Putative binding sites of CHREBP and p65/RELA on mouse *Acsl1*. Arrows denote the region amplified by qPCR. B) Chromatin was immunoprecipitated from BMDMs differentiated in HG using an antibody against CHREBP and isotype-matched IgG control. C) Chromatin was immunoprecipitated from BMDMs differentiated in HG and stimulated with LPS (10ng/mL) for 1 hour using an antibody against p65/RELA along with isotype-matched IgG control. Percent of precipitated DNA compared to total input DNA is shown. The data are means with an error bar representing the spread of the mean from two independent experiments.

We then performed ChIP for CHREBP and p65/RELA to determine whether these transcription factors occupy the *Acsl1* promoter in BMDMs in HG and upon LPS stimulation. Indeed, CHREBP and p65/RELA occupied the *Acsl1* upstream regulatory region containing the predicted motifs for these factors (Fig 6). We did not find any binding of p65/RELA above IgG on *Acsl1* in the absence of LPS treatment (not shown). Thus, CHREBP and p65/RELA occupy the *Acsl1* promoter in BMDMs under conditions of hyperglycemia and inflammation.

## Discussion

ACSL1 is part of a family of enzymes that promotes the thioesterification of long-chain fatty acids to form acyl-CoAs for use in lipid synthetic or degradative pathways [2, 3], and in monocytes/macrophages ACSL1 uses arachidonic acid as a specific substrate to produce prostaglandin E2 (PGE2) to promote inflammation [5]. The expression of *Acsl1* mRNA is induced by inflammation and hyperglycemia [3]. However, the mechanisms underlying the upregulation of *Acsl1* mRNA have remained enigmatic. This study shows a strong association of *Acsl1* expression with CHREBP in hyperglycemia and NF-kappa B under inflammatory conditions.

CHREBP increases *Acsl1* mRNA expression in HG in murine macrophages and human monocytes. This is reflected in the glucose and CHREBP-dependent induction of *Acsl1* mRNA and is consistent with the increased expression of *Acsl1* observed in monocytes and macrophages in humans and mice under hyperglycemic conditions [5].

In the current report, acute inflammatory stimulation by LPS in macrophages promotes a robust induction of *Acsl1* mRNA and is suppressed by an NF-kappa B inhibitor. This is consistent with recent reports showing increased *ACSL1* mRNA in septic patients [32] and patients with acute myocardial infarction compared to controls [14], which may reflect an inflammatory response from necrotic tissue as a result of ischemia. We also show that the induction of *Acsl1* expression by inflammation is enhanced by hyperglycemia, indicative of an interplay between the CHREBP and NF-kappa B. In support of this, we show increased *Acsl1*-luciferase reporter activity upon co-expression of CHREBP and NF-kappa B compared to either factor alone. Thus, the induction of *Acsl1* mRNA can occur by CHREBP or NF-kappa B depending on the stimulus (e.g., HG or LPS), but is further augmented in the presence of both HG and LPS.

**Fig 7:**
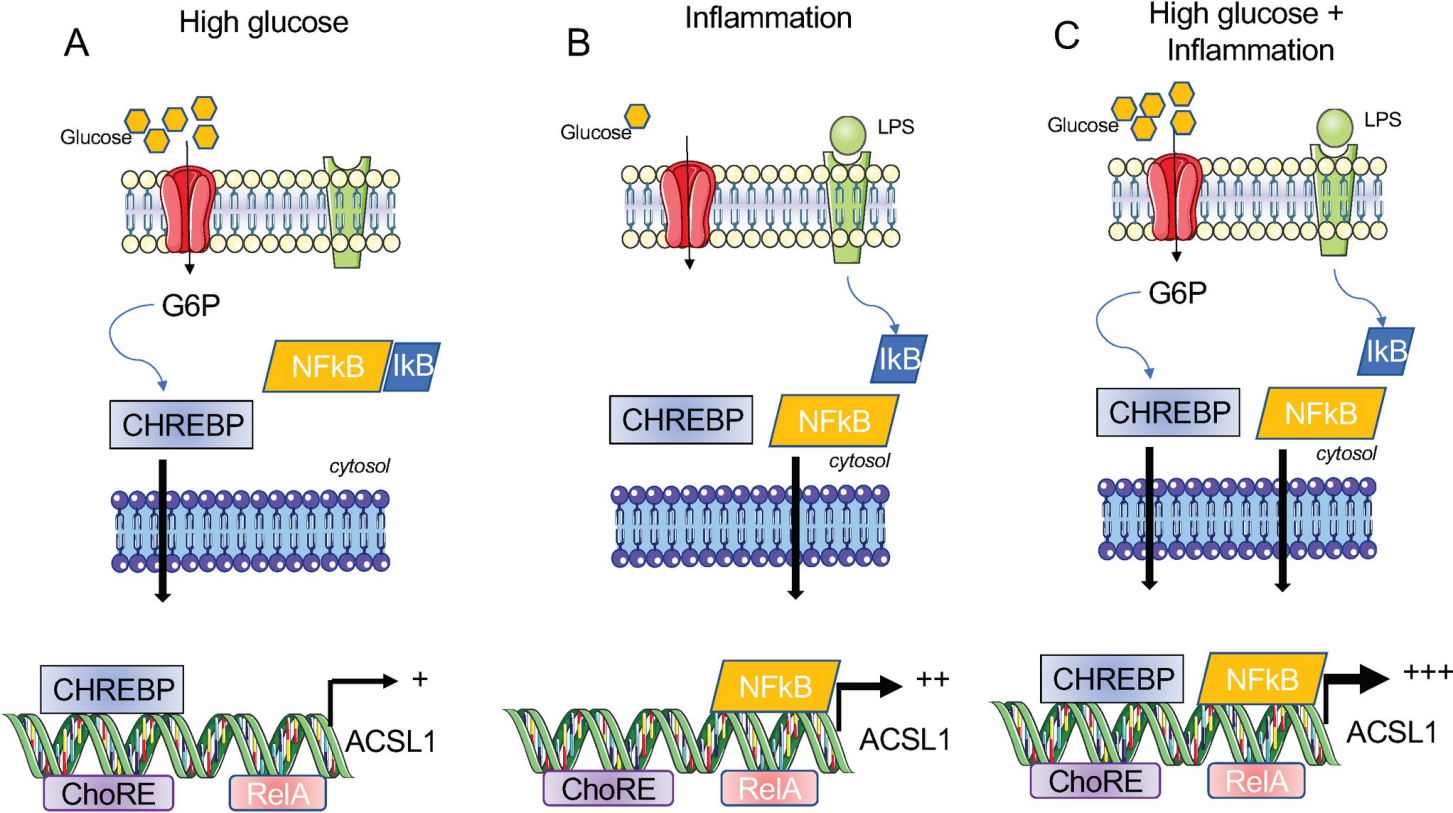
Model for HG and inflammation-induced *Acsl1* expression by CHREBP and NF-kappa B in macrophages. A) We propose that in high glucose, CHREBP is depressed, and active nuclear CHREBP promotes the expression of *Acsl1*. B) Under inflammatory conditions that activate the NF-kappa B via dismissal of the inhibitory protein I kappa B (IkB) that prevents NF-kappa B nuclear transport, *Acsl1* transcription is induced. C) *Acsl1* expression can be further increased by high glucose and inflammatory stimuli via CHREBP and NF-kappa B.

Based on these findings, we propose a model whereby in macrophages under conditions of HG, *Acsl1* expression is primarily driven by CHREBP (Fig 7A). Upon exposure to inflammatory stimuli, *Acsl1* expression is induced by NF-kappa B (Fig 7B), which can be further enhanced by CHREBP in HG (Fig 7C). Although we favor a model that supports the direct regulation of *Acsl1* expression by CHREBP and NF-kappa B, we do not rule out the indirect effects of these factors on the regulation of *Acsl1*. Regardless, our studies reveal that hyperglycemia and inflammation and their related transcription factors CHREBP and NF-kappa B foster *Acsl1* expression in macrophages to support the production of acyl-CoA derivatives. Our findings are not limited to mouse models. *ACSL1* expression was elevated in human monocytes upon HG and LPS stimulation, supporting our study’s relevance to humans. In summary, the current report provides a molecular framework to understand the mechanisms that explain the pro-atherogenicity of ACSL1 in diabetes.

## Supporting information

S1 Fig Increased *Acsl1* mRNA expression in BMDMs upon acute HG treatment. BMDMs were differentiated in normal glucose (NG; 5.5 mM), high glucose (HG; 25mM), or NG and then switched to HG for 24 hours. *Acsl1* mRNA expression relative to cyclophilin A was determined by qPCR. *Acsl1* mRNA expression is shown as fold change with the NG treated sample set to 1. The data are means with error bars representing the spread of the means from two replicate experiments.
(PDF)

S2 Fig Increased nuclear localization of CHREBP in macrophages in HG. A) BMDMs were differentiated under NG and HG conditions. Cytoplasmic and membrane proteins were isolated, and a western blot was performed using an anti-CHREBP antibody to determine the abundance and subcellular localization of CHREBP protein. Tubulin and Histone H3 served as controls for cytoplasmic and nuclear fractions, respectively. B) BMDMs were differentiated under NG and HG conditions. The cells were grown on coverslips and stained for CHREBP and DAPI to visualize the nucleus. The images were obtained using Leica SP5 Confocal Microscope at 63X magnification.
(PDF)

S3 Fig Kinetics of *Acsl1* induction by LPS. BMDMs were differentiated in NG and treated with LPS (10ng/ml). RNA was isolated at the indicated times, and *Tnf*, *Il6*, and *Acsl1* mRNA were determined by qPCR relative to cyclophilin A. The data presented are means ± standard errors of the means of two independent experiments.
(PDF)

S4 Fig ACSL1 mRNA induction upon hyperglycemia and LPS stimulation in human monocytes. Human monocytes from healthy donors were differentiated under NG and HG conditions. Cells were either left untreated or treated with LPS (10ng/mL) for 24 hours. Total RNA was isolated, and human ACSL1 mRNA was measured by qPCR relative to 18S RNA and shown as fold change. NG in the absence of an LPS treatment sample was set to 1. The data presented are means ± standard errors of the means (n = 4); the p-value was calculated using one-way ANOVA. (p < 0.05; **p < 0.01; and ***p < 0.001).
(PDF)

S5 Fig Predicted CHREBP and NF-kappa B DNA binding motifs upstream of the mouse and human ACSL1 genes. The Eukaryotic Promoter Database tool was used to predict putative binding sites for CHREBP (red rectangles) and NFκB (blue triangles) 2 kb upstream of the mouse and human promoter using the transcription factor motifs present in the Jaspar database. The p-value used for the motif prediction was p<0.001.
(PDF)
