# Supplementary material for: Transcriptional regulation of Acsl1 by CHREBP and NF-kappa B in macrophages during hyperglycemia and inflammation
Source: PLoS One. 2022 Sep 2;17(9):e0272986. doi: 10.1371/journal.pone.0272986 (PMC9439225; doi:10.1371/journal.pone.0272986)
Supplement: S1 Fig — BMDMs were differentiated in normal glucose (NG; 5.5 mM), high glucose (HG; 25mM), or NG and then switched to HG for 24 hours. Acsl1 mRNA expression relative to cyclophilin A was determined by qPCR. Acsl1 mRNA expression is shown as fold change with the NG treated sample set to 1. The data are means with error bars representing the spread of the means from two replicate experiments. (PDF) [file pone.0272986.s001.pdf]

Supplementary Figure 1

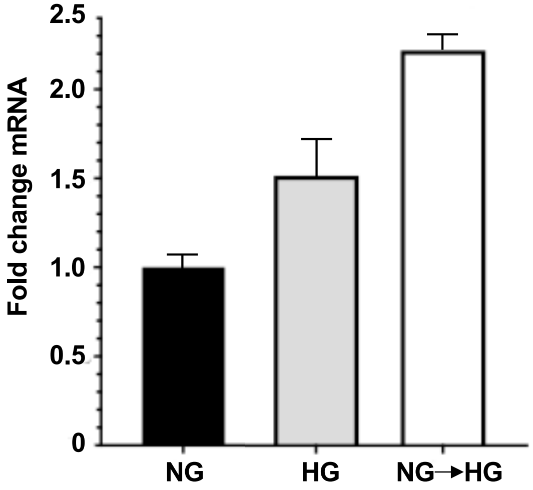

S1 Fig. Increased *Acsl1* mRNA expression in BMDMs upon acute HG treatment.
BMDMs were differentiated in normal glucose (NG; 5.5 mM), high glucose (HG; 25mM) , or in NG, and then switched to HG for 24 hours. *Acsl1* mRNA expression relative to cyclophilin A was determined by qPCR. *Acsl1* mRNA expression is shown as fold change with the NG treated sample set to 1. The data are means with error bar representing the spread of the means from two replicate experiments.
