# Supplementary material for: Transcriptional regulation of Acsl1 by CHREBP and NF-kappa B in macrophages during hyperglycemia and inflammation
Source: PLoS One. 2022 Sep 2;17(9):e0272986. doi: 10.1371/journal.pone.0272986 (PMC9439225; doi:10.1371/journal.pone.0272986)
Supplement: S2 Fig — A) BMDMs were differentiated under NG and HG conditions. Cytoplasmic and membrane proteins were isolated, and a western blot was performed using an anti-CHREBP antibody to determine the abundance and subcellular localization of CHREBP protein. Tubulin and Histone H3 served as controls for cytoplasmic and nuclear fractions, respectively. B) BMDMs were differentiated under NG and HG conditions. The cells were grown on coverslips and stained for CHREBP and DAPI to visualize the nucleus. The images were obtained using Leica SP5 Confocal Microscope at 63X magnification. (PDF) [file pone.0272986.s002.pdf]

Supplementary Figure 2

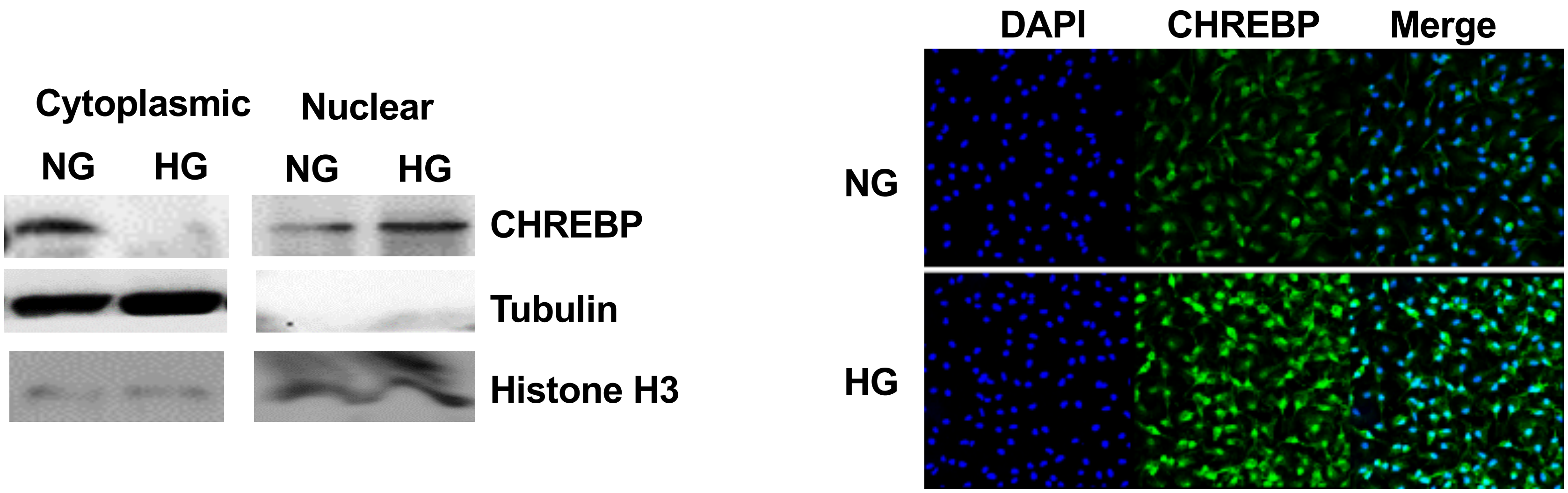

S2 Fig. Increased nuclear localization of CHREBP in macrophages in hyperglycemia.
A) BMDMs were differentiated under NG and HG conditions. Cytoplasmic and membrane proteins were isolated and western blot performed using an anti-CHREBP antibody to determine the abundance and subcellular localization of CHREBP protein. Tubulin and Histone H3 served as controls for cytoplasmic and nuclear fractions, respectively. B) BMDMs were differentiated under NG and HG conditions. The cells were grown on cover slips and stained for CHREBP and DAPI to visualize the nucleus. The images were obtained using Leica SP5 Confocal Microscope at 63X magnification.
