# Supplementary material for: Transcriptional regulation of Acsl1 by CHREBP and NF-kappa B in macrophages during hyperglycemia and inflammation
Source: PLoS One. 2022 Sep 2;17(9):e0272986. doi: 10.1371/journal.pone.0272986 (PMC9439225; doi:10.1371/journal.pone.0272986)
Supplement: S3 Fig — BMDMs were differentiated in NG and treated with LPS (10ng/ml). RNA was isolated at the indicated times, and Tnf, Il6, and Acsl1 mRNA were determined by qPCR relative to cyclophilin A. The data presented are means ± standard errors of the means of two independent experiments. (PDF) [file pone.0272986.s003.pdf]

Supplementary Figure 3

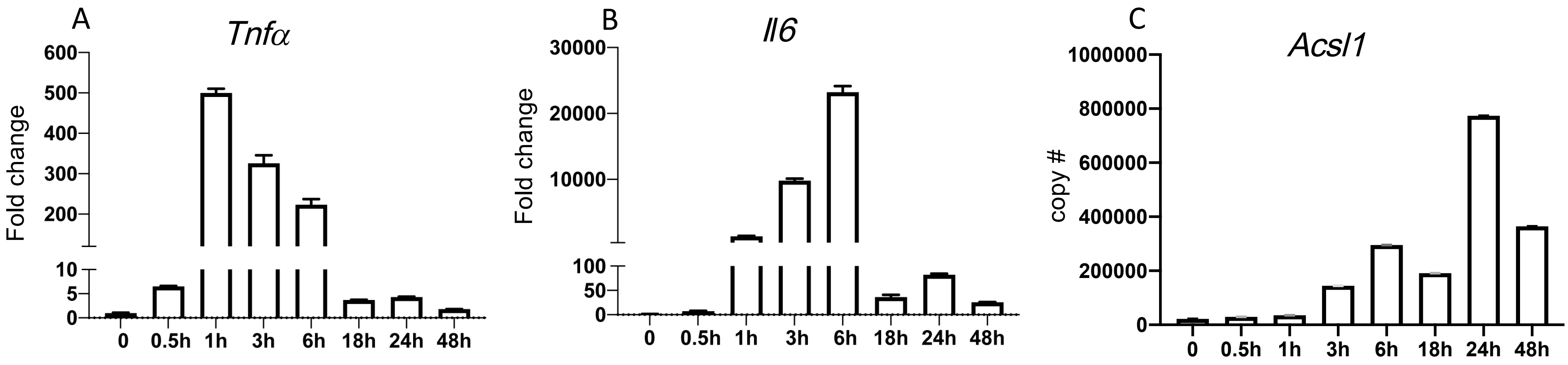

S3 Fig. Kinetics of *Acsl1* induction by LPS treatment.
BMDMs were differentiated in NG and treated with LPS (10ng/ml). RNA was isolated at the indicated times, cDNA was synthesized, and *Tnf*, *Il6*, and *Acsl1* mRNA were determined by qPCR relative to cyclophilin A. The data presented are means ± standard errors of the means of two independent experiments.
