# Supplementary material for: Transcriptional regulation of Acsl1 by CHREBP and NF-kappa B in macrophages during hyperglycemia and inflammation
Source: PLoS One. 2022 Sep 2;17(9):e0272986. doi: 10.1371/journal.pone.0272986 (PMC9439225; doi:10.1371/journal.pone.0272986)
Supplement: S4 Fig — Human monocytes from healthy donors were differentiated under NG and HG conditions. Cells were either left untreated or treated with LPS (10ng/mL) for 24 hours. Total RNA was isolated, and human ACSL1 mRNA was measured by qPCR relative to 18S RNA and shown as fold change. NG in the absence of an LPS treatment sample was set to 1. The data presented are means ± standard errors of the means (n = 4); the p-value was calculated using one-way ANOVA. (p < 0.05; **p < 0.01; and ***p < 0.001). (PDF) [file pone.0272986.s004.pdf]

## Supplementary Figure 4

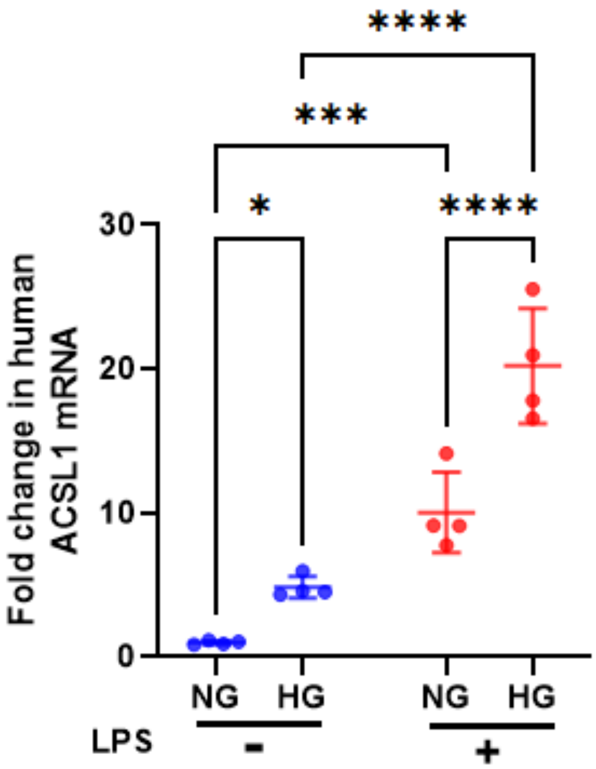


**S4 Fig. ACSL1 mRNA induction upon high glucose and LPS stimulation in human monocytes.**
Human monocytes from healthy donors were differentiated under NG and HG conditions. Cells were either left untreated or treated with LPS (10ng/mL) for 24 hours. Total RNA was isolated and human ACSL1 mRNA was measured by qPCR relative to 18S RNA, and shown as fold change. NG in the absence of LPS treatment sample was set to 1. The data presented are means ± standard errors of the means (n=4); the p-value was calculated using one way ANOVA. ($p < 0.05$; **$p < 0.01$; and ***$p < 0.001$).
