# Supplementary material for: Transcriptional regulation of Acsl1 by CHREBP and NF-kappa B in macrophages during hyperglycemia and inflammation
Source: PLoS One. 2022 Sep 2;17(9):e0272986. doi: 10.1371/journal.pone.0272986 (PMC9439225; doi:10.1371/journal.pone.0272986)
Supplement: S5 Fig — The Eukaryotic Promoter Database tool was used to predict putative binding sites for CHREBP (red rectangles) and NFκB (blue triangles) 2 kb upstream of the mouse and human promoter using the transcription factor motifs present in the Jaspar database. The p-value used for the motif prediction was p<0.001. (PDF) [file pone.0272986.s005.pdf]

Supplementary Figure 5

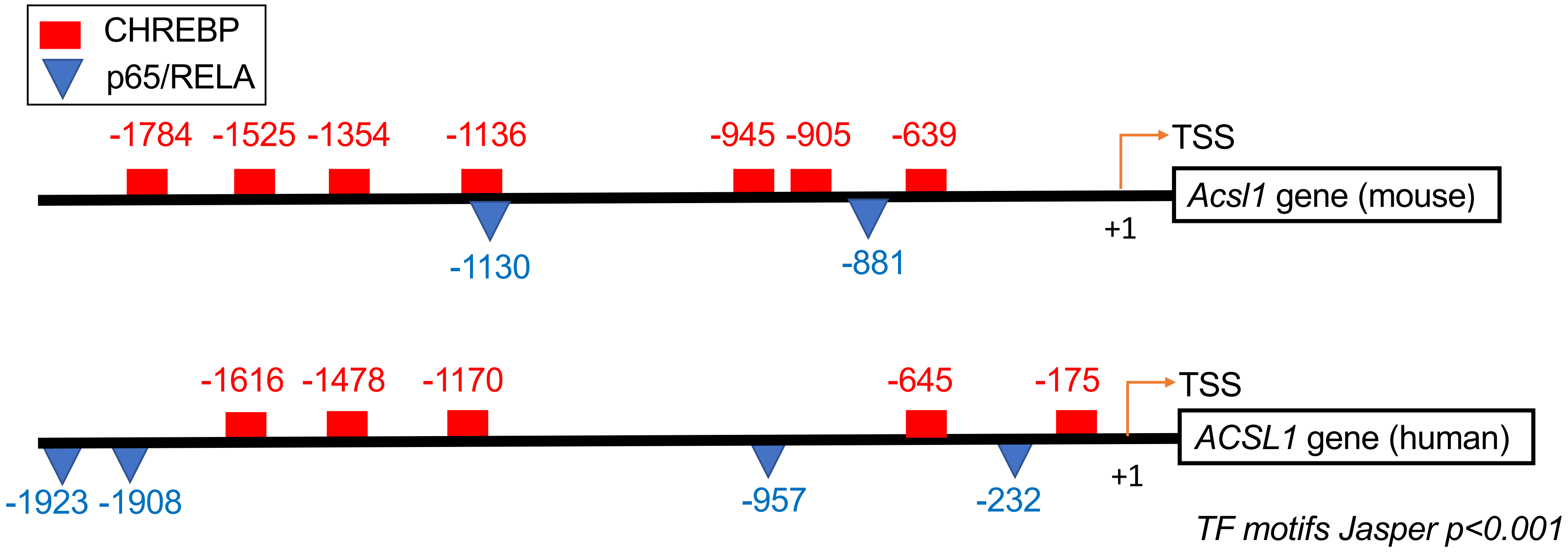

S5 Fig. Predicted CHREBP and NF-kappa B DNA binding motifs upstream of the mouse and human ACSL1 genes.
The Eukaryotic Promoter Database tool was used to predict putative binding sites for CHREBP (red rectangles) and NFκB (blue triangles) 2Kb upstream of the mouse and human promoter using the transcription factor motifs present in the Jaspar database. The P-value used for the motif prediction was p<0.001.
